# Plasma free amino acid profiling as metabolomic diagnostic and prognostic biomarker in paediatric cancer patients: a follow-up study

**DOI:** 10.1007/s00726-020-02910-8

**Published:** 2020-11-11

**Authors:** Anna Synakiewicz, Anna Stanislawska-Sachadyn, Malgorzata Sawicka-Zukowska, Grazyna Galezowska, Joanna Ratajczyk, Anna Owczarzak, Malgorzata Skuza, Lidia Wolska, Teresa Stachowicz-Stencel

**Affiliations:** 1 Department of Pediatrics, Hematology and Oncology, Medical University of Gdansk, 7 Debinki Street, 80-211, Gdansk, Poland; 2 Department of Molecular Biotechnology and Microbiology, Gdansk University of Technology, Gdansk, Poland; 3 Department of Pediatric Oncology and Hematology, Medical University of Bialystok, Bialystok, Poland; 4 Department of Environmental Toxicology, Medical University of Gdansk, Gdansk, Poland; 5 Department of Clinical Nutrition, Medical University of Gdansk, Gdansk, Poland; 6 Diagnostic Imaging Department, The Polish Red Cross Maritime Hospital, Gdynia, Poland

**Keywords:** Amino acid profile, Metabolomics, Cancer, Tumor biomarkers

## Abstract

Amino acids (AAs) play a crucial role in cancer cell metabolism. Levels of 22 plasma AAs at the time of diagnosis and after treatment were established among 39 pediatric cancer patients and 33 healthy children. Glutamic acid levels decreased and tryptophan levels increased during treatment. Cancer patients presented significantly lower levels of glutamine and leucine post-treatment while levels of 12 other AAs were higher comparing to controls. Results suggest that plasma free AA profile may serve as a prognostic biomarker.

**Electronic supplementary material:**

The online version of this article (doi:10.1007/s00726-020-02910-8) contains supplementary material, which is available to authorized users.

## Introduction

Amino acids (AAs) as metabolic key regulators of many cell pathways are essential for differentiation, growth and maintenance of immunological balance of human cells.

A cachectic oncological patient commonly presents hypermetabolism with intensified processes of lipolysis, fatty acid oxidation and gluconeogenesis, as well as protein catabolism (Bernstein and Ortiz 2005; Giovannucci 1999). Studies on amino acids carried out on animal cancer models have shown an increased protein catabolism in muscle cells with a simultaneous decrease in their synthesis and an increase in total protein turnover compared to control group (Kawamura et al. 1982).

Our previous research concerning plasma-free amino acid (PFAA) profile in pediatric oncological patients at the time of diagnosis showed significant differences in PFAA levels between pediatric cancer patients and control group (Synakiewicz et al. 2017). In the current research, we additionally included post-treatment data to find out the severity of changes and its tendency to recur to normal values. We made an attempt to establish if PFAAs may serve as prognostic biomarkers.

## Materials and methods

### Patients

Thirty-nine pediatric cancer patients, including 18 with hematological malignancies and 21 with solid tumors, who were diagnosed and treated in the Department of Pediatrics, Hematology and Oncology, Medical University of Gdansk, Poland and in the Department of Pediatric Oncology and Hematology, Medical University of Bialystok, Poland from 2012 to 2014, were enrolled in the follow-up study (Table 1).

**Table 1:** Characteristics of study population. In-time changes of amino-acids levels for two time points: diagnosis and post-treatment. Comparison of amino acids levels from cases after treatment and healthy controls

	Cases time 1 (diagnosis)		Cases time 3 (post-treatment), follow-up		Healthy controls			
Age at blood collection	Median (q1–q3), *N*	Mean, StDev	Median (q1–q3), *N*	Mean, StDev	Median (q1–q3), *N*	Mean, StDev		
All	8 (2–16), 39	9.14, 6.45	8.7 (3.5–16.7), 39	9.99, 6.52	13 (9–16), 33	11.91, 4.87		
Hematologic malignancies	14 (6–16), 18	11.83, 5.26	15.2 (6.5–17.1), 18	12.73, 5.41				
Solid tumours	4 (2–12), 21	6.83, 6.59	5.3 (2.4–13.6), 21	7.65, 6.59				

Amino acid^a^							*P* value^b^ cases before vs. after treatment	*P *value^c^ cases after treatment vs. controls
Aspartic acid (Asp)	102.1 (17.6–123.4), 39	82.8, 82.2	103 (4.3–119.1), 39	73.2, 59.7	22.9 (15.8–32.05), 32	24.6, 16.1	0.5553	0.1139
Glutamic acid (Glu)	112.1 (82–169.9), 39	132.6, 73.9	78.5 (60.7–108.2), 39	123.0, 206.9	86.8 (69.1–123.8), 33	96.5, 40.7	0.004	0.4185
Asparagine (Asn)	79.5 (33.2–104.1), 38	72.6, 49.5	94.65 (15.1–102.1), 38	69.9, 45.9	21.5 (14.8–29.6), 33	25.5, 19.6	0.6182	0.0060
Glutamine (Gln)	357.4 (269.8–473.2), 39	434.2, 329.1	306.0 (201.7–414.2), 39	321.3, 155.5	741.2 (375.1–1106.4), 33	777.0, 469.3	0.1657	< .0001
Serine (Ser)	151.3 (70.3–213.3), 39	146.7, 74.5	150.3 (59.1–201), 39	140.7, 80.0	78.9 (55.2–101.5), 33	79.2, 35.8	0.5693	0.0023
Histidine (His)	89.4 (47.8–150.9), 39	117.1, 94.7	71.2 (42.0–15.0), 39	107.2, 96.4	127.5 (77.8–168.5), 33	139.6, 89.8	0.5279	0.0308
Citrulline/glycine (Cit/Gly)	164.6 (136.8–208.7), 39	231.3, 263.4	175.6 (148.8–569.6), 39	383.2, 386.2	134.4 (80.1–168.6), 33	142.2, 84.3	0.0429	0.0003
Arginine (Arg)	97.85 (67.7–151.3), 38	128.4, 87.9	115.15 (74.3–200.5), 38	140.4, 92.9	85.95 (64.8–118.0), 32	104.6, 69.5	0.983	0.0941
Threonine (Thr)	142.5 (90.6–194.3), 39	155.6, 86.3	128.0 (66.4–206.9), 39	140.9, 85.1	118.6 (87.65–175.85), 32	135.6, 69.4	0.5012	0.7429
Taurine (Tau)	65.3 (44.8–147.7), 38	107.0, 90.6	54.5 (42.0–85.6), 38	75.4, 53.2	98.9 (38.05–148.25), 32	102.1, 64.4	0.2729	0.0855
Alanine (Ala)	175.5 (96.1–323.4), 39	205.8, 137.1	189.4 (77.8–290.7), 39	224.7, 173.1	85.15 (67.4–198.8), 32	132.2, 98.9	0.4335	0.0238
Tyrosine (Tyr)	173.0 (126.2–223.7), 39	182.0, 101.3	170.6 (133.9–230.7), 39	216.6, 154.8	111.8 (73.9–286.3), 33	172.0, 142.4	0.4417	0.0505
GABA	74.6 (37.9–86.6), 39	69.2, 38.1	77.1 (45.0–85.5), 39	68.3, 9.9	34.9 (28.9–42.3), 33	36.6, 11.3	0.8695	< .0001
Tryptophan (Trp)	104.3 (25.9–164.8), 39	100.4, 67.2	150.9 (72.9–170.2), 39	130.6, 52.5	22.1 (14.5–52.0), 33	33.4, 26.8	0.0163	< .0001
Methionine (Met)	44.1 (20.2–94.1), 39	62.8, 9.3	84.6 (25.3–92.9), 39	65.8, 34.2	19.2 (16.9–22.6), 33	19.5, 5.5	0.5834	< .0001
Valine (Val)	202.8 (138.3–248.0), 39	201.6, 89.7	234.3 (171.0–265.7), 39	226.2, 59.6	157.7 (131.5–185.3), 33	158.5, 50.9	0.0809	< .0001
Phenylalanine (Phe)	109.4 (64.3–142.6), 39	106.4, 51.1	115.1 (74.7–133.5), 39	107.6, 39.1	46.2 (31.3–69.1), 33	64.1, 83.7	0.8803	< .0001
Isoleucine (Ile)	109.3 (55.4–143.2), 39	102.3, 53.0	109.3 (73.2–136.1), 39	112.0, 55.3	45.0 (33.6–55.9), 33	45.1, 8.4	0.612	< .0001
Leucine (Leu)	202.9 (113.7–273.1), 39	225.2, 56.8	174.2 (100.5–240.3), 39	235.6, 404.7	351.6 (140.9–543.2), 33	348.2, 215.6	0.2821	0.0059
Ornithine (Orn)	69.35 (55.05–128.05), 36	102.3, 90.9	94.7 (68.4–151.95), 36	130.5, 92.3	60.9 (55.5–103.3), 30	81.6, 49.0	0.1253	0.0048
Lysine (Lys)	104.9 (82.4–132.5), 37	121.4, 59.0	144.2 (90.9–175.2), 37	144.8, 68.2	130.8 (101.3–162.3), 33	133.9, 42.7	0.1589	0.7022

^a^Amino acids concentration: µmol/l

^b^Wilcoxon signed ranked test

^c^Wilcoxon ranked-sum test

### Inclusion criteria

We included children aged between 1 month and 18 years with confirmed neoplastic disease. The patients showed no signs of infection.

### Control group

Thirty-three healthy children were recruited (23 males and 10 females), aged between 2 and 18 years (mean age 11.9 years, median age 13 years).

### Laboratory analysis

Blood samples were withdrawn from the subjects at the time of diagnosis and after successful treatment, approximately 1 to 6 months after the end of therapy. The average duration of treatment was about 2 years.

Measurements of amino acid plasma concentrations were performed using high-performance liquid chromatography with fluorometric detection.

All analyses were performed in the laboratory of the Department of Clinical Nutrition and Department of Environmental Toxicology; Medical University of Gdansk, Poland.

Particular description of the laboratory methods was given in our previously published article (Synakiewicz et al. 2017).

### Statistical analysis

Descriptive analyses of the study population included medians and percentiles as well as means and standard deviations for continuous variables and proportions for categorical variables. In the follow-up analyses, we only included the cases with available levels of PFAAs both at the time of diagnosis and after treatment. The chi-squared test was used to determine differences in categorical variables between the oncological cases and the controls. The Wilcoxon rank-sum tests were used to determine differences in PFAA concentrations, comparing the cases to the healthy subjects. The differences in amino acid levels between pairs of observations, specifically the patients before and after treatment, were determined using the Wilcoxon signed rank test. Tests were two-tailed and *P* values ≤ 0.05 were considered statistically significant.

Statistical analyses were calculated using SAS 9.4 (NC, USA). XLStat (Addinsoft) programme was used to generate plots.

## Results

**Fig. 1:**
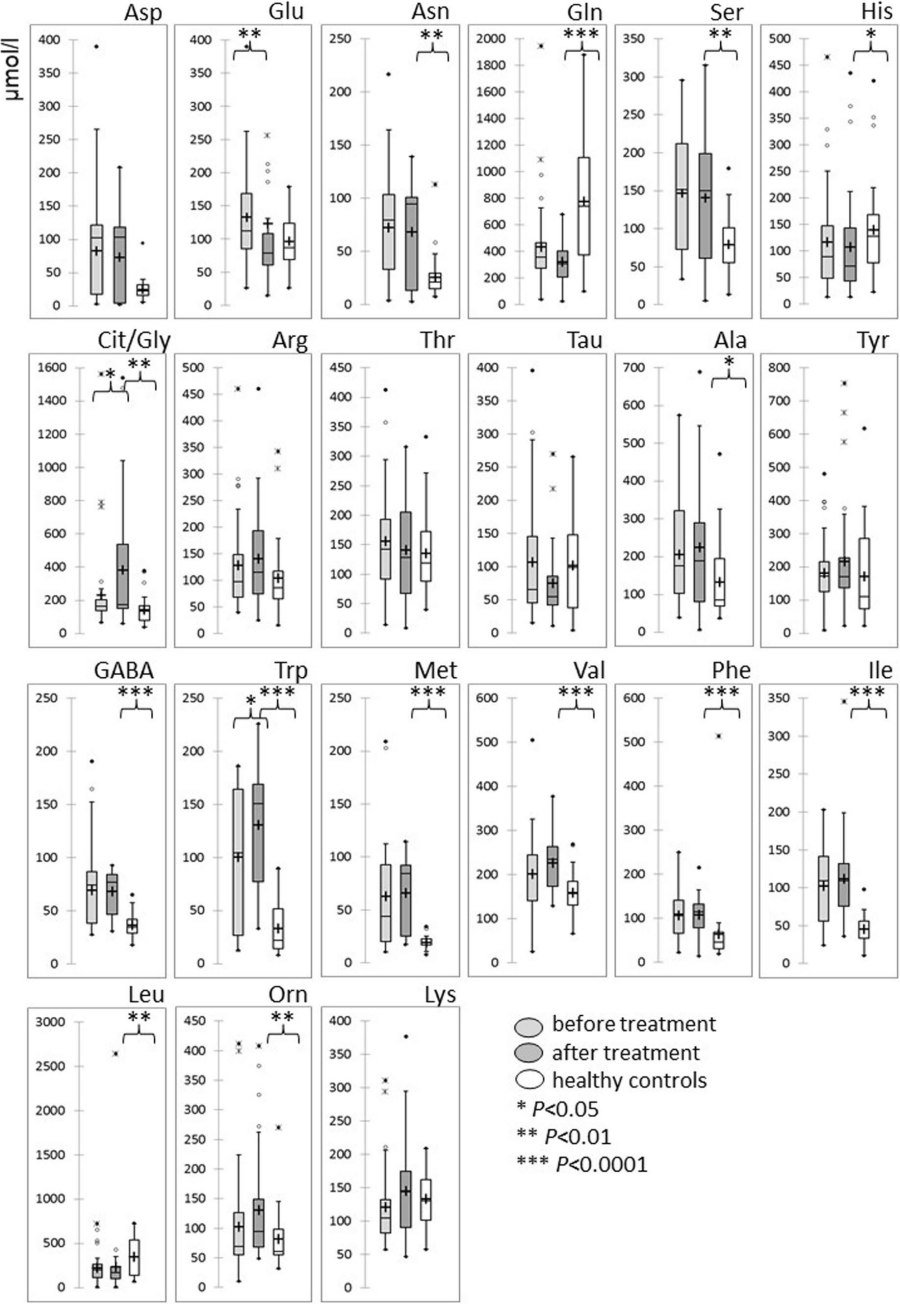
Changes of amino acid levels from 39 pediatric patients at the time of diagnosis and post-treatment. Comparison to amino acid levels in 33 healthy controls

Plasma levels of AA from the patients who successfully finished treatment and healthy controls were compared. Significantly lower levels of plasma Gln (*P* < 0.0001), His (*P* = 0.0308) and Leu (*P* = 0.0059) were observed in the cancer patients in comparison to the controls. The patients showed significantly higher levels of Asn (*P* = 0.0060), Ser (*P* = 0.0023), Cit/Gly (*P* = 0.0003), Ala (*P* = 0.0238), GABA (*P* < 0.0001), Trp (*P* < 0.0001), Met (*P* < 0.0001), Val (*P* < 0.0001), Phe (*P* < 0.0001), Ile (*P* = 0.0059) and Orn (*P* = 0.0048) (Table 1, Fig. 1).

Analyses performed to determine changes in PFAAs before and after treatment revealed a significant decrease in plasma Glu levels (*P* = 0.004) and a significant increase in Trp (*P* = 0.0163) and Cit/Gly (*P* = 0.0429) levels after treatment. Levels of other analyzed PFAAs did not show any change in the course of treatment (Table 1, Fig. 1).

Analyses were performed allowing for cancer type, specifically: solid tumors and hematologic malignancies. Due to low numbers of patients, those results are preliminary and we list only the most interesting findings (Supplementary Table 1, Supplementary Table 2). Significantly lower levels of plasma His (*P* = 0.0061) were found among cases with solid tumors, but not hematologic malignancies, when compared to controls. Levels of Asn (*P* = 0.044), Ser (*P* = 0.0013), Ala (*P* = 0.0115) and Orn (*P* = 0.0069) were higher among cases with solid tumors, but not hematologic malignancies, when compared to controls.

Levels of Glu decreased during treatment were significantly different among patients diagnosed both with solid tumors (*P* = 0.0286) and with hematologic malignancies (*P* = 0.0737), although the later difference was border-significant. Difference in Trp levels before and after treatment remained significant among patients diagnosed with solid tumors (*P* = 0.0167) but not among those who were diagnosed with hematologic malignancies. In-time changes in Cit/Gly levels did not reach significance in analyses with regard to cancer type.

## Discussion

PFAAs may partly reflect tumour-induced alterations in protein metabolism. A study by Lai et al. (2005) has focused on PFAA profile variability in cancer patients. The researchers have assumed that at the early stages of disease amino acid turnover rate is increased due to tumor cell hypermetabolism. The origin of tumour cells may also be relevant, as malignant transformation occurs to varying degrees depending on the cell type and this may affect host protein metabolism (Kubota et al. 1992; Cascino et al. 1995; Hensley et al. 2013). Thus, PFAA levels have been proposed as an additional tool in diagnosis and monitoring of malignant disease. In this follow-up study, we have evaluated how PFAA levels change during treatment in a group of cancer pediatric patients.

Cancer patients have shown higher plasma Trp levels in comparison to healthy subjects both before (Synakiewicz et al. 2017) and after successfully finished treatment (*P* < 0.0001). Moreover, Trp levels were elevated after treatment *(P* = 0.0163). These observations were unexpected since in a large study conducted on adults opposite results were obtained (Onesti et al. 2019) indicating poorer prognosis in low-Trp-level patients. Thus, further studies on protein degradation with Trp release in oncologic pediatric patients are warranted.

We observed significantly lower Gln levels in cancer patients comparing to controls, both at the beginning (Synakiewicz et al. 2017) and after treatment, (*P* < 0.0035 and *P* < 0.0001, respectively). Gln has been widely studied in terms of malignancy and its role in cancer metabolism (Hensley et al. 2013). Neoplastic cells are consumers of Gln used for cell proliferation and serving as carbon and nitrogen supplier (Dunphy et al. 2018; Hosios et al. 2016). In addition to being an important fuel for proliferating cells, Gln participates in control of gene expression, and is also involved in the activation and repair of intracellular signaling pathways (Neu et al. 1996). A significant decrease in plasma Gln levels has already been observed in case of cancer patients (Kobayashi et al. 2013; Miyagi et al. 2011). An increased uptake of Gln by neoplastic cells may be caused by its interdependence on tricarboxylic acid cycle, redox reactions and mTOR activity. Interestingly, significantly higher levels of Glu in our patients have been determined at the beginning of the treatment in comparison to control group (*P* < 0.0399) (Synakiewicz et al. 2017), while levels of Glu decreased at the course of therapy in our study population (*P* = 0.004), which is consistent with observations from studies on colorectal and breast cancer patients (Okamoto et al. 2009; Barnes et al. 2014). Many studies have emphasized that due to metabolic re-programming malignant cells use more effective transport of Gln to create Glu allowing to produce energy via lactate and glutaminase overexpression (Medina 2001; DeBerardinis et al. 2008; Erickson and Cerione 2010).

Analyses of plasma samples from patients with tumours of breast, stomach and thyroid gland by Gu et al. (2015) have presented apparent differences in PFAA behaviour in different tumour type patients relative to healthy controls. Post-surgery levels of Asp returned to normal in breast cancer and gastric cancer patients, while Ala levels returned to normal in breast cancer patients. In our pediatric patients, levels of Glu returned to normal post-treatment. Interestingly, Gu et al. (2015) studied proliferative effect of several AAs and have found that Ala, Arg, Asn and Cys promoted proliferation of breast cancer cells, Cys has stimulated proliferation of gastric neoplastic cells, while Ala and Glu have induced apoptosis of the latter. PFAA profile changes have also been demonstrated in hepatocellular carcinoma patients (Raouf et al. 2016). It showed significant increase of Met, Tyr, Orn, Cyt, Gly, Phe, Ala, Glu, Pro plasma levels and a decrease in Leu level obtained from untreated cancer patients, which was consistent with our results.

While discussing PFAA profile changes, it is worth considering potential factors affecting behavior of those molecules. A therapy with l-asparaginase is well known and routinely used for treatment of acute lymphoblastic leukaemia (ALL) in pediatric patients, as these cells depend on blood serum asparagine due to lack of asparagine synthetase (ASNS). Application of this drug results in the reduction of Asn in blood serum with simultaneous depletion of Gln which mediates in asparagine synthesis (Covini et al. 2012). Differences in Asn levels we observed were close to significant in our haematological group of patients, among whom were those with ALL.

Disturbances in levels of AAs may be merely a matter of oncological patients’ nutritional status, their individual predisposition or may possibly depend on the stage of the disease or the type of cancer.

## Conclusion

Significant alterations in PFAA profile observed in a study group of pediatric oncological patients with different types of malignancies indicate a dependency between a cancer process and disturbances in amino acid kinetics. Thus, PFAA profiling may serve as an early diagnostic biomarker of a neoplastic process. In this study, we have compared PFAA levels before and after successful treatment of pediatric oncological patients. Our results indicate that PFAA profile may become not only a prognostic tool but also a monitoring value. However, it requires further research that would include patients in clinical remission and comparing them to cases with treatment failure.

## Electronic supplementary material

Below is the link to the electronic supplementary material.

Supplementary material 1 (DOCX 30 KB)
