# Supplementary material for: Plasma free amino acid profiling as metabolomic diagnostic and prognostic biomarker in paediatric cancer patients: a follow-up study
Source: Amino Acids. 2020 Nov 11;53(1):133–8. doi: 10.1007/s00726-020-02910-8 (PMC7822799; doi:10.1007/s00726-020-02910-8)
Supplement: Supplementary file 1 — Supplementary material 1 (DOCX 30 KB) [file 726_2020_2910_MOESM1_ESM.docx]

**Plasma free amino acids profiling as metabolomic diagnostic and prognostic biomarkers in paediatric cancer patients – a follow-up study**

**SUPPLEMENTARY MATERIAL**

Anna Synakiewicz^1^, Anna Stanislawska-Sachadyn^2^, Malgorzata Sawicka-Zukowska^3^, Grazyna Galezowska^4^, Joanna Ratajczyk^4^, Anna Owczarzak^5^, Malgorzata Skuza^6^, Lidia Wolska^4^, Teresa Stachowicz-Stencel^1*^

^1^ Department of Pediatrics, Hematology and Oncology, Medical University of Gdansk, Poland

^2^ Department of Molecular Biotechnology and Microbiology, Gdansk University of Technology

^3^ Department of Pediatric Oncology and Hematology, Medical University of Bialystok, Poland

^4^ Department of Environmental Toxicology, Medical University of Gdansk, Poland

^5^ Department of Clinical Nutrition, Medical University of Gdansk, Poland

^6^ Diagnostic Imaging Department, The Polish Red Cross Maritime Hospital, Gdynia, Poland

Corresponding author: MD, PhD, Assoc.Prof. Teresa Stachowicz-Stencel

Department of Pediatrics, Hematology and Oncology

Medical University of Gdansk

7 Debinki Street, 80-211, Gdansk, Poland

Tel: +48 58 349 28 80 Fax: +48 58 3492847

e-mail: tsten@gumed.edu.pl

Supplementary Table 1. In-time changes of amino-acids levels for two time points: diagnosis and post-treatment. Patients diagnosed with hematologic malignancies. Comparison of amino acids levels from cases after treatment and healthy controls.

| **Amino acid^1^** | **Cases**  **Time 1 (diagnosis)** | | **Cases**  **Time 3 (post-treatment)** | | **Healthy controls** | | ***P*-*value*^2^**  **Cases**  **Before vs. after treatment** | ***P-value*^3^**  **Cases after treatment vs. controls** |
| --- | --- | --- | --- | --- | --- | --- | --- | --- |
|  | **Median (q1-q3),**  **N** | **Mean, StDev** | **Median (q1-q3),**  **N** | **Mean, StDev** | **Median (q1-q3),**  **N** | **Mean, StDev** |  |  |
| Aspartic acid (Asp) | 102.75 (24.1-119.4),  18 | 75.66, 51.89 | 61.1 (5.4-125.5),  18 | 66.75, 61.31 | 22.9 (15.8-32.05),  32 | 24.6,  16.1 | 0.3247 | 0.5808 |
| Glutamic acid (Glu) | 145.9 (74.2-200.1),  18 | 152.34, 91.09 | 76.05 (46.5-130.7),  18 | 156.04, 300.40 | 86.8 (69.1-123.8),  33 | 96.5,  40.7 | 0.0737 | 0.4172 |
| Asparagine (Asn) | 93.5 (54.7-104.1),  17 | 80.88, 36.70 | 91 (11.5-98.2),  17 | 60.67, 46.01 | 21.5 (14.8-29.6),  33 | 25.5,  19.6 | 0.0638 | 0.1166 |
| Glutamine (Gln) | 340.45 (277.5-458.2), 18 | 373.53, 147.22 | 335.65 (201.7-390.8),  18 | 318.71, 134.60 | 741.2 (375.1-1106.4), 33 | 777.0,  469.3 | 0.2837 | 0.0012 |
| Serine (Ser) | 147.25 (70.3-211.2), 18 | 141.02, 77.27 | 127 (51.6-196.4),  18 | 124.24, 71.63 | 78.9 (55.2-101.5),  33 | 79.2,  35.8 | 0.4423 | 0.0907 |
| Histidine (His) | 96.45 (47.8-154.9),  18 | 108.41, 71.39 | 102.3 (55.2-180.2),  18 | 141.35, 126.06 | 127.5 (77.8-168.5),  33 | 139.6,  89.8 | 0.5509 | 0.5121 |
| Citrulline/Glycine (Cit/Gly) | 161.3 (144.5-171.5), 18 | 166.49, 52.30 | 171.55 (150.3-505.4), 18 | 373.39, 398.62 | 134.4 (80.1-168.6),  33 | 142.2,  84.3 | 0.1297 | 0.0097 |
| Arginine (Arg) | 105.2 (67.7-136.4), 17 | 116.65, 63.55 | 167.9 (99.6-202.3),  17 | 157.81, 74.79 | 85.95 (64.8-118.0),  32 | 104.6,  69.5 | 0.2435 | 0.0122 |
| Threonine (Thr) | 164.3 (115.5-217.9), 18 | 171.96, 73.16 | 134.2 (61.3-230.2),  18 | 154.27, 89.75 | 118.6 (87.65-175.85), 32 | 135.6,  69.4 | 0.5798 | 0.5015 |
| Taurine (Tau) | 56.55 (47.9-82.9),  18 | 74.59, 46.34 | 67.75 (52.5-126.2),  18 | 90.07, 66.63 | 98.9 (38.05-148.25), 32 | 102.1,  64.4 | 0.2462 | 0.3675 |
| Alanine (Ala) | 199.95 (96.1-320.3), 18 | 208.06, 141.06 | 149.75 (72.3-299.7), 18 | 214.36, 182.88 | 85.15 (67.4-198.8),  32 | 132.2,  98.9 | 0.6397 | 0.2674 |
| Tyrosine (Tyr) | 174.95 (142-223.7), 18 | 193.13, 104.01 | 176.2 (142.1-323.5), 18 | 218.61, 156.36 | 111.8 (73.9-286.3),  33 | 172.0,  142.4 | 0.8650 | 0.2464 |
| GABA | 81 (40.4-86.6),  18 | 72.03, 38.93 | 74.65 (45-82.6),  18 | 65.11, 20.81 | 34.9 (28.9-42.3),  33 | 36.6,  11.3 | 0.5798 | <.0001 |
| Tryptophan (Trp) | 147.8 (23.9-168.5),  18 | 101.23, 73.62 | 124.85 (65.7-173.7), 18 | 116.44, 57.09 | 22.1 (14.5-52.0),  33 | 33.4,  26.8 | 0.3927 | <.0001 |
| Methionine (Met) | 84 (18.8-91.6), 18 | 59.09, 38.49 | 85.55 (20.3-94.5), 18 | 62.65, 37.89 | 19.2 (16.9-22.6),  33 | 19.5,  5.5 | 0.6397 | 0.0002 |
| Valine (Val) | 206.6 (114.1-240),  18 | 183.38, 82.35 | 234.35 (171-274.7),  18 | 228.65, 61.46 | 157.7 (131.5-185.3), 33 | 158.5,  50.9 | 0.0665 | 0.0007 |
| Phenyloalanine (Phe) | 130.1 (72-158.5),  18 | 118.69, 50.17 | 121.8 (63.2-140.8),  18 | 108.12, 50.31 | 46.2 (31.3-69.1),  33 | 64.1,  83.7 | 0.3927 | 0.0005 |
| Isoleucine (Ile) | 104.65 (46.1-139.4), 18 | 94.87, 52.25 | 117.7 (52.5-136.1),  18 | 113.66, 74.07 | 45.0 (33.6-55.9),  33 | 45.1,  18.4 | 0.5226 | <.0001 |
| Leucine (Leu) | 200.5 (114.6-254.5), 18 | 205.34, 117.29 | 120.7 (94.1-226.6),  18 | 162.88, 93.91 | 351.6 (140.9-543.2),  33 | 348.2,  215.6 | 0.4171 | 0.0064 |
| Ornitine (Orn) | 58.2 (43.5-121.85),  16 | 95.53, 99.50 | 85.85 (71.65-96.5),  16 | 89.63, 28.87 | 60.9 (55.5-103.3),  30 | 81.6,  49.0 | 0.7057 | 0.0590 |
| Lysine (Lys) | 98.5 (79.4-116.6),  18 | 102.24, 33.52 | 155.95 (130.5-188.3), 18 | 153.77, 46.71 | 130.8 (101.3-162.3), 33 | 133.9,  42.7 | 0.0066 | 0.1379 |

^1^amino acids concentration: µmol/l

**^2^**Wilcoxon signed ranked test

**^3^**Wilcoxon ranked-sum test

Supplementary Table 2. In-time changes of amino-acids levels for two time points: diagnosis and post-treatment. Patients diagnosed with solid tumours. Comparison of amino acids levels from cases after treatment and healthy controls.

| **Amino acid^1^** | **Cases**  **Time 1 (diagnosis)** | | **Cases**  **Time 3 (post-treatment)** | | **Healthy controls** | | ***P*-*value*^2^**  **Cases**  **Before vs. after treatment** | ***P-value*^3^**  **Cases after treatment vs. controls** |
| --- | --- | --- | --- | --- | --- | --- | --- | --- |
|  | **Median (q1-q3),**  **N** | **Mean, StDev** | **Median (q1-q3),**  **N** | **Mean, StDev** | **Median (q1-q3),**  **N** | **Mean, StDev** |  |  |
| Aspartic acid (Asp) | 31 (7.7-125.4),  21 | 88.90, 102.27 | 104.1 (3.9-111.2), 21 | 78.76, 59.22 | 22.9 (15.8-32.05),  32 | 24.6,  16.1 | 0.9333 | 0.0497 |
| Glutamic acid (Glu) | 103.7 (91.7-123.8),  21 | 115.75, 51.73 | 78.5 (64.8-107.3), 21 | 94.77, 52.77 | 86.8 (69.1-123.8),  33 | 96.5,  40.7 | 0.0286 | 0.5967 |
| Asparagine (Asn) | 38 (23.5-102.1),  21 | 65.88, 57.83 | 94.8 (21.2-104.3), 21 | 77.30, 45.64 | 21.5 (14.8-29.6),  33 | 25.5,  19.6 | 0.6023 | 0.0044 |
| Glutamine (Gln) | 377.2 (224.9-527.9), 21 | 486.18, 425.62 | 241.3 (214.7-414.2), 21 | 323.59, 174.77 | 741.2 (375.1-1106.4), 33 | 777.0,  469.3 | 0.3339 | 0.0004 |
| Serine (Ser) | 165 (91.5-214.8),  21 | 151.57, 73.68 | 156 (87.3-206.6),  21 | 154.72, 85.67 | 78.9 (55.2-101.5),  33 | 79.2,  35.8 | 0.9599 | 0.0013 |
| Histidine (His) | 89.4 (50-145.3),  21 | 124.49, 112.24 | 62.4 (39.5-100.9), 21 | 77.90, 46.75 | 127.5 (77.8-168.5),  33 | 139.6,  89.8 | 0.1245 | 0.0061 |
| Citrulline/Glycine (Cit/Gly) | 175.8 (134.8-214.2), 21 | 286.94, 349.88 | 179.8 (148.8-569.6), 21 | 391.55, 384.91 | 134.4 (80.1-168.6),  33 | 142.2,  84.3 | 0.1756 | 0.0010 |
| Arginine (Arg) | 90.5 (71-170.3),  21 | 137.91, 104.19 | 85.2 (64.1-170.5), 21 | 126.33, 104.93 | 85.95 (64.8-118.0),  32 | 104.6,  69.5 | 0.2842 | 0.7243 |
| Threonine (Thr) | 141.3 (66.2-157.8),  21 | 141.53, 95.70 | 128 (67.9-164.7),  21 | 129.42, 81.28 | 118.6 (87.65-175.85), 32 | 135.6,  69.4 | 0.7756 | 0.9351 |
| Taurine (Tau) | 97.15 (41.9-211.75), 20 | 136.08, 110.35 | 49.5 (41.8-78.05), 20 | 62.11, 33.98 | 98.9 (38.05-148.25), 32 | 102.1,  64.4 | 0.0153 | 0.0594 |
| Alanine (Ala) | 159.6 (108.4-323.4), 21 | 203.85, 137.14 | 213.4 (114.7-272.2), 21 | 233.49, 168.37 | 85.15 (67.4-198.8),  32 | 132.2,  98.9 | 0.5789 | 0.0115 |
| Tyrosine (Tyr) | 154.8 (115.5-209.3), 21 | 172.52, 100.49 | 168.8 (133.9-222.1), 21 | 214.88, 157.29 | 111.8 (73.9-286.3),  33 | 172.0,  142.4 | 0.4477 | 0.0445 |
| GABA | 51.4 (37.9-86.6),  21 | 66.80, 38.19 | 77.1 (63.2-86.5),  21 | 71.02, 19.10 | 34.9 (28.9-42.3),  33 | 36.6,  11.3 | 0.4477 | <.0001 |
| Tryptophan (Trp) | 97.1 (39-160.8),  21 | 99.74, 63.01 | 150.9 (138.9-168), 21 | 142.66, 46.24 | 22.1 (14.5-52.0),  33 | 33.4,  26.8 | 0.0167 | <.0001 |
| Methionine (Met) | 37.9 (20.7-94.9),  21 | 66.06, 57.76 | 84 (30-90.2),  21 | 68.45, 31.43 | 19.2 (16.9-22.6),  33 | 19.5,  5.5 | 0.7756 | <.0001 |
| Valine (Val) | 198.2 (156.5-282.9), 21 | 217.28, 94.62 | 232.6 (175.7-260.4), 21 | 224.01, 59.37 | 157.7 (131.5-185.3), 33 | 158.5,  50.9 | 0.4896 | 0.0005 |
| Phenyloalanine (Phe) | 84 (64.3-121),  21 | 95.82, 50.64 | 115.1 (91.4-126.9), 21 | 107.23, 27.45 | 46.2 (31.3-69.1),  33 | 64.1,  83.7 | 0.2688 | <.0001 |
| Isoleucine (Ile) | 113.9 (62.3-146.6),  21 | 108.75, 54.06 | 105.7 (97.7-128.1), 21 | 110.65, 33.88 | 45.0 (33.6-55.9),  33 | 45.1,  18.4 | 0.9866 | <.0001 |
| Leucine (Leu) | 211.7 (109-283.7),  21 | 242.26, 185.28 | 177.6 (150.4-240.3), 21 | 297.99, 542.94 | 351.6 (140.9-543.2),  33 | 348.2,  215.6 | 0.6023 | 0.0655 |
| Ornitine (Orn) | 84.1 (62.05-128.05), 20 | 107.78, 85.55 | 134.8 (65.8-247.55), 20 | 163.19, 111.83 | 60.9 (55.5-103.3),  30 | 81.6,  49.0 | 0.1231 | 0.0069 |
| Lysine (Lys) | 120 (85-183.2), 19 | 139.59, 71.95 | 125.2 (77.6-167.2), 19 | 136.37, 84.19 | 130.8 (101.3-162.3), 33 | 133.9,  42.7 | 0.5153 | 0.4070 |

^1^amino acids concentration: µmol/l

**^2^**Wilcoxon signed ranked test

**^3^**Wilcoxon ranked-sum test
